# Intercellular Interactomics of Human Brain Endothelial Cells and Th17 Lymphocytes: A Novel Strategy for Identifying Therapeutic Targets of CNS Inflammation

**DOI:** 10.1155/2011/175364

**Published:** 2011-06-13

**Authors:** Arsalan S. Haqqani, Danica B. Stanimirovic

**Affiliations:** Institute for Biological Sciences, National Research Council, Ottawa, ON, Canada K1A 0R6

## Abstract

Leukocyte infiltration across an activated brain endothelium contributes to the neuroinflammation seen in many neurological disorders. Recent evidence shows that IL-17-producing T-lymphocytes (e.g., Th17 cells) possess brain-homing capability and contribute to the pathogenesis of multiple sclerosis and cerebral ischemia. The leukocyte transmigration across the endothelium is a highly regulated, multistep process involving intercellular communications and interactions between the leukocytes and endothelial cells. The molecules involved in the process are attractive therapeutic targets for inhibiting leukocyte brain migration. We hypothesized and have been successful in demonstrating that molecules of potential therapeutic significance involved in Th17-brain endothelial cell (BEC) communications and interactions can be discovered through the combination of advanced membrane/submembrane proteomic and interactomic methods. We describe elements of this strategy and preliminary results obtained in method and approach development. The Th17-BEC interaction network provides new insights into the complexity of the transmigration process mediated by well-organized, subcellularly localized molecular interactions. These molecules and interactions are potential diagnostic, therapeutic, or theranostic targets for treatment of neurological conditions accompanied or caused by leukocyte infiltration.

## 1. Leukocyte Infiltration in CNS Disorders

The central nervous system (CNS) has long been regarded as an “immune privileged” organ, being both immunologically inert and immunologically separated from the peripheral immune system [1]. Current data, however, indicates that the CNS is both immune competent and actively interactive with the peripheral immune system [2]. In physiological conditions, a limited number of peripheral immune cells cross the blood-brain barrier (BBB) and enter the CNS in a process called “immune surveillance” [1]. Many neurological diseases are associated with a much higher rate of leukocyte trafficking into the CNS, resulting in leukocyte infiltration and leukocyte-mediated neuronal damage. CNS inflammation is a major contributor to the diverse forms of brain injury seen in cerebral ischemia, multiple sclerosis, cerebral infection, and epilepsy [3–5]. 

A growing body of recent evidence shows that infiltration of a subset of IL-17-producing T-lymphocytes into the CNS contributes to the pathogenesis of multiple sclerosis, and cerebral ischemia. In multiple sclerosis these cells are CD4+ T helper 17 (Th17) lymphocytes that have CNS-homing properties and mediate the immune response directed at the myelin sheath. Gyulveszi and coworkers recently demonstrated that the CNS tropism of Th17 cells is driven by IL23, since T cells defective in IL-23 signaling fail to accumulate in the CNS in the mouse model of experimental autoimmune encephalomyelitis (EAE) [6]. These findings are consistent with the report by Prat and colleagues, showing that IL-23-stimulated Th17 lymphocytes promote BBB disruption *in vitro* and in the EAE, efficiently penetrate the BBB, kill neurons, and promote further CNS inflammation through CD4+ lymphocyte recruitment [7]. 

In experimental ischemic stroke, T-lymphocytes are localized to the infarction boundary zones [8] and contribute to the development of secondary inflammatory brain injury [9]. More recently, Shichita et al. have shown that in mice subjected to a transient middle cerebral artery occlusion an initial infiltration of IL-23-producing macrophages is followed by subsequent recruitment/activation of IL-17-producing *γδ*T lymphocytes [10]. These T cells concomitantly increase downstream proinflammatory and neurotoxic factors and the infarct size after focal cerebral ischemia [10]. Thus, as in multiple sclerosis, IL17-producing T cells activated by IL-23 are believed to “home-in” towards the CNS and induce injury during cerebral stroke.

## 2. Molecular Mechanisms of Leukocyte Migration through the Endothelial Layer

The endothelial lining of brain capillaries exhibits a specialized phenotype, commonly referred to as the blood-brain barrier (BBB). These endothelial cells (ECs) function as a restrictive gate to control the composition of extracellular fluid in the central nervous system (CNS), selectively restricting and/or controlling the access of blood-borne molecules to the brain [11]. The brain capillary endothelium exhibits unique anatomical and biochemical features, including tight junctions (TJ) that form a physical barrier for a majority of hydrophilic molecules larger than 500 Da, low pinocytic activity, and the *polarized* expression of transporters that control both brain influx and efflux of molecular substrates [11]. 

The *luminal* surface of BEC (i.e., the side accessible to the blood) contains a thick glycocalyx, which is enriched in proteins and glycoproteins involved in key BBB functions, including the transport of solutes and macromolecules, BEC permeability, vasoreactivity, and interactions with circulating cells and platelets [12–15]. Under inflammatory conditions, luminal adhesion molecules in the glycocalyx directly interact with leukocytes during the highly-regulated, multistep transmigration process involving tethering and rolling, activation, arrest, and diapedesis [16, 17]. These steps are mediated by surface adhesion molecules and cytokines on both leukocytes and BEC and the avidity of interactions among different intercellular interacting molecular pairs. The initial low-affinity contacts, leading to tethering and rolling, slow down the flowing leukocytes and are mediated by the binding of selectins on the glycocalyx of BEC to their paired ligands on leukocytes. Firm adhesion (arrest) of the leukocytes is mediated by the binding of cell adhesion molecules (CAMs) on EC to their respective integrins on the leukocyte surface. ICAM1 and VCAM1 are two well-known CAMs that are overexpressed on brain EC in response to inflammatory insults. ICAM1 can pair with either *α*L*β*2 (LFA1, CD11a/CD18) or *α*M*β*2 (Mac1, CD11b/CD18) integrins, while VCAM1 interacts with *α*4*β*1 (VLA4, CD49d/CD29) integrin on leukocytes [18]. 

Leukocyte diapedesis from the luminal surface of BEC to its abluminal side is the last step in the transmigration process and is the least well understood. The step has been traditionally believed to take place through the paracellular route, that is, through the TJ of BEC [19]. A number of intercellular interacting pairs (IIPs), including JAM1-JAM1, JAM1-LFA1, PECAM1-PECAM1, and CD99-CD99, have been identified between BEC TJ and leukocytes [20]. However, the paracellular migration route has been repeatedly challenged by histological and electron microscopy studies demonstrating that leukocytes can migrate through the transcellular pathway, that is through the BEC themselves, leaving the TJ morphologically intact [17, 18, 21]. While it is likely that the leukocyte migration occurs through both pathways [17], the details of the molecular events involved in either of the pathways remain limited.

## 3. Therapies against Leukocyte Migration into the Brain: Successes and Failures

Inhibiting the interactions between the migrating leukocytes and the BEC is an attractive therapeutic approach for inhibiting tissue inflammation. A target is usually a molecule that is essential in the transmigration process and is easily accessible to therapy. The IIPs discussed above are some of the key players in different stages of the leukocyte transmigration process and are also blood-accessible molecules, that is, present on either the surface of leukocytes or the luminal membranes of BEC. Blocking antibodies against the selected leukocyte-brain BEC IIPs have already been developed and used in preclinical studies and clinical trials. 

Two IIPs have been primarily targeted for the inhibition of CNS inflammation: VCAM1-VLA4 interactions or ICAM1-LFA1 interactions. Blocking antibodies and other drug molecules against the *α*4-integrin part of VLA4 have been shown to inhibit or reverse the EAE in various animal models (summarized in [18]). These findings led to the development of a humanized monoclonal anti-*α*4-integrin antibody, Natalizumab, which was evaluated in two randomized, double-blind, placebo-controlled trials in patients with multiple sclerosis. The results of the trials demonstrated a large benefit for patients taking Natalizumab, including a 42% reduction in the risk of sustained disability progression and a 68% reduction in relapse rate [22]. The drug was initially approved by FDA in 2004, but was subsequently withdrawn after it was linked with few cases of progressive multifocal leukoencephalopathy (PML) when combined with immunomodulatory treatment [22, 23]. However, after a review of safety information, the drug was returned to the market in 2006 because its clinical benefits outweighed the risks involved. While Natalizumab has proven to be very successful in controlling the disease, some limitations due to side effects have been reported, including elevated lymphocyte, basophil, and eosinophil counts, with 5% of drug recipients showing circulating nucleated erythrocytes [23]; these side effects are likely due to widespread functions of *α*4 integrin in the hematopoietic system [18, 23].

Blocking antibody therapies targeting ICAM1 and LFA1 have also been developed. *In vitro *studies using these antibodies have consistently shown that the ICAM1-LFA1 interaction is important for T-cell adhesion and diapedesis across brain endothelium (summarized in [18]). However, these therapies have produced highly conflicting results in CNS inflammation *in vivo*. While some studies have shown a beneficial outcome of blocking either ICAM1 or LFA1, others could not find a significant inhibition or even showed worsening of the outcome and early mortality in the EAE animal models [18, 24]. Furthermore, clinical trails using humanized anti-LFA1 (Rovelizumab, LeukArrest) and anti-ICAM1 (Enlimomab) antibodies have shown lack of efficacy or serious side effects. Another anti-VLA1 antibody (Efalizumab) has been approved for the therapy of psoriasis, a non-CNS inflammatory disorder [25]. Thus, while the role of ICAM1-LFA1 interaction is controversial in CNS inflammation, this interaction plays an important role (similar to VCAM1-VLA4 interaction) in peripheral, non-CNS inflammation.

## 4. Unmet Needs and the Hypothesis

### 4.1. Discovery of Novel, Specific Targets

Currently, only a limited number of molecules on leukocytes and the BBB are known or known to interact. For example, ICAM1-LFA1 and VCAM1-VLA4 pairs have been known for more than two decades and are still pursued as the main targets for inhibiting interactions between endothelium and leukocytes in the brain. Targeting these interactions alone does not provide complete protection, suggesting that other adhesion molecules may “compensate” when these interactions are blocked [26]. From problems associated with the functional redundancy of leukocyte-BEC molecular interactions and the inhibition of peripheral immune system function with current treatments, an important need has emerged to discover molecules specifically involved in the adhesion and diapedesis of specific subsets of leukocytes through the BBB that are less important in the overall immune system competency. With emerging evidence of the role of the IL-23-induced IL-17-producing T cells in the pathogenesis of multiple sclerosis and cerebral ischemia, there is a need for identification of more specific molecules on these subpopulations of leukocytes, which confer their brain-tropism, to develop more cell-selective targets. Further, there is a need to identify cell-specific (and human-specific) molecules as targets on brain EC to minimize non-specific side effects of current drugs (described above). We propose that human brain EC and human brain tropic T-cells should become key models to discover novel targets implicated in neuroinflammatory cell recruitment and migration.

## 5. Hypothesis

Using advanced methods that combine membrane and submembrane proteomics and glycoproteomics with methods of in silico interactomics, a novel set of intercellular interactions between human brain endothelial cells and human CNS-homing T cells, can be identified and exploited as therapeutic targets for preventing brain inflammation caused by recruitment of peripheral inflammatory cells.

## 6. Testing the Hypothesis

**Figure 1:**
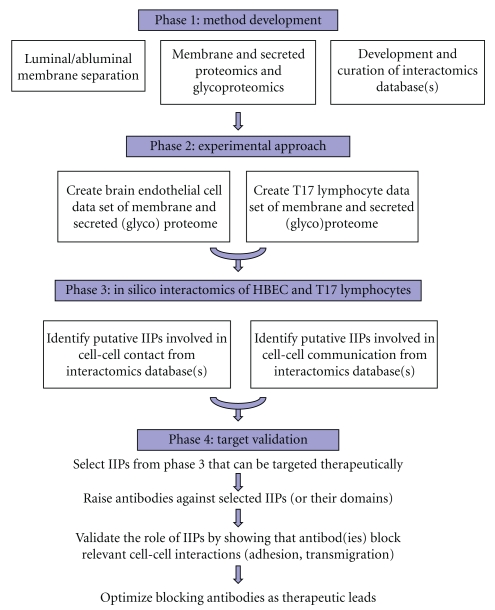
Schematic flowchart of the proposed approach to identify protein-protein interactions between Th17 lymphocytes and brain endothelial cells that are functionally implicated in the recruitment of inflammatory/immune cells across the blood-brain barrier.

The flow chart of proposed strategy to test the above hypothesis is shown in Figure 1. The *first phase* of the strategy includes the development of appropriate methods for membrane and submembrane proteomics and glycoproteomics and appropriate model system(s) for testing the hypothesis, including the generation and curation of database(s) of relevant known protein-protein and protein-carbohydrate interactions. The *second phase* involves the application of membrane and submembrane proteomic methods to the selected model system to identify protein and glycoprotein changes induced in endothelial cells and CNS-tropic lymphocytes under pathological conditions (i.e., inflammation or ischemia). In the *third phase*, intercellular interacting pairs (IIPs) between brain BEC and CNS-tropic lymphocytes, with potential roles in leukocyte-endothelium contact or communication (i.e., interactions among secreted proteins and cell surface proteins), are identified using *in silico *interactomics. Finally, *validation* of the role of identified IIPs in leukocyte adhesion/transmigration is accomplished using *in vitro* assays and biological readouts. Elements and phases of this strategy, and preliminary results obtained in method and approach development, are described in detail below.

### 6.1. Phase 1: Development and Validation of Methods

#### 6.1.1. Membrane and Submembrane Proteomics Methods

Proteins present on cell membranes, facing the extracellular environment, are the main site of contacts between BEC and leukocytes during adhesion and diapedesis. The contact site on BEC is mainly the luminal membrane (glycocalyx), which consists of a complex mixture of proteins, glycoproteins, and other molecules. Since BEC have “polarized” membranes, that is, molecules on the luminal and abluminal membranes are different, there is also a need to couple submembrane fractionation with proteomics to identify the differences between the various fractions. Analyzing membrane molecules has usually been difficult, especially using proteomics. Traditional 2D gel-based proteomic methods lack the sensitivity, reproducibility, and the ability to analyze the complete spectrum of membrane proteins, due to inherent limitation of the technology (summarized in [27]). A quantitative and reproducible method for analyzing glycosylated proteins on a global scale has been lacking. 

Over the last 5 years, technological growth in the proteomics field has led to the development of advanced nanoLC-MS-based systems that are composed of highly reproducible and sensitive nanoflow ultra HPLC systems (e.g., nanoAquity), coupled with sensitive and high mass-accuracy MS instruments (e.g., Orbitrap) [28]. We and others have also developed bioinformatics software that analyze nanoLC-MS data, allowing the quantification of thousands of proteins and glycoproteins in multiple biological samples [29]. In addition, the recent development of hydrazide capture technology [30, 31] has enabled selective enrichment of glycoproteins from cells and tissues for large-scale identification, using nanoLC-MS-based quantitative proteomics. Recently, we also described methods to enrich for various membrane fractions (e.g., luminal, abluminal) from human BEC and other cells, and coupled this with hydrazide capture and quantitative nanoLC-MS-based proteomics [28, 30]. This combination of submembrane isolation methods and advanced proteomics and glycoproteomics technology is needed to discover novel proteins and glycoproteins on the surfaces of BEC and T cells.

#### 6.1.2. Intercellular Interactomics Data Base(s)

Proteomics and other genomics methods generate overwhelming data sets of molecules that become difficult to follow up. Since validating entire “lists” becomes costly and time consuming, a single-factor/reductionist approach is often undertaken to select (or “cherry pick”) a couple of molecules for further evaluation. As a result, a significant portion of disease-implicated molecules are simply overlooked. Thus, there is a need for development and application of alternative methodology to identify the overlooked molecules, which may include novel and more specific targets. Systems biology is the field of biology that aims to provide a more “holistic” view by examining the network of interactions between multiple molecules, pathways, cells, and characteristics of the tissue, as they converge to determine the disease implication of the molecules [32]. Since molecular interactions are the fundamentals of systems biology, a large amount of effort has been invested into generating protein-interaction databases through large-scale experimental interactomics and curation of the scientific literature [33]. We propose to use the curated protein-protein interaction databases and systems biology approaches, to identify which molecules actually interact between BEC and brain-specific lymphocytes. This *in silico *interactomics methodology has a potential to identify novel IIPs as therapeutic targets for CNS inflammation.

Protein interactions have traditionally been measured using immunoprecipitation and yeast two-hybrid systems. However, high-throughput techniques have also been developed that identify affinity pull-down complexes using advanced proteomics [34], and systematically constructed double-knockout strains in yeast have proven to be useful for constructing a large-scale view of molecular interaction networks [35]. As a result, a number of publicly available protein-protein interaction databases currently exist, including BIND, Human Protein Reference Database, HiMAP, BioGRID, and EcoCyc. Utilizing these existing databases and datasets, we reconstructed an in-house database consisting of more than a million molecular interactions. For the current study, we limited the interactions to immunoprecipitation and affinity pull-down assays in mammalian systems to reduce the incidence of false interactions. The resulting mammalian protein-protein interaction database (mPPI-db) consists of more than 200,000 nonredundant interactions, and was used for the discovery of novel IIPs between human brain endothelial cells (BEC) and lymphocytes using the experimental approaches described below.

### 6.2. Phase 2: Experimental Models and Their Validation

#### 6.2.1. Activated Human BEC

Brain endothelium undergoes significant changes in response to inflammation and becomes more receptive to interactions towards immune cells. This “activation” of the endothelium is characterized by molecular and physical changes in the luminal glycocalyx. Because proposed studies are focused on BEC-lymphocyte interactions relevant for human disease, to test the hypothesis we used the hCMEC/D3 human brain endothelial cell line as a stable human *in vitro* model of the BBB [36]. The cells were grown in EBM-2 media (Lonza, Walkersville, MD) supplemented with 2% FBS and were activated under serum-free conditions using various inflammatory insults, including TNF*α*/INF*γ*, IL-1*β*, or simulated ischemia/reperfusion conditions as previously described [26, 37, 38]. Although lymphocyte transmigration process *in vivo* occurs in postcapillary venules, there are currently no *in vitro* BBB models that specifically distinguish endothelium from capillaries and venules; most models, including the one used in these studies are mixed population of endothelial cells originating from both. The hCMEC/D3 have been demonstrated to be valid for studies of BBB function, responses of brain endothelium to inflammatory and infectious stimuli, and the interaction of brain endothelium with lymphocytes or other cells [26, 36]. From the activated cells, proteins from luminal (apical) and abluminal (basolateral) membranes and from cellular and secreted fractions were isolated using recently described methods [28], enriched for glycoproteins using hydrazide capture [30], and analyzed using nanoLC-MS-based quantitative proteomics to identify differentially expressed molecules [39]. In total, more than 4500 unique proteins and glycoproteins were identified in the human BEC, with about 650 present on each the luminal and abluminal membranes. In addition, about 25–30% of all the proteins responded significantly to the inflammatory insults, several of which were well-known indicators of BEC activation. Upregulation of surface adhesion molecules on luminal membranes (including ICAM1, VCAM1) changes in several TJ proteins [19] and integrins, including *β*1, *β*2, *α*4, *α*L, *α*M were observed. A number of proteins involved in *intra*cellular signaling and downstream pathways were also detected, including those involved in the recruitment of the “transmigratory cup” machinery [20] to the luminal membranes, for example, ezrin, moesin, radixin, and other cytoskeletal proteins, suggesting that the cells have been “primed” for the anticipated process of leukocyte adhesion. Furthermore, about 20% of the proteins were associated with BEC-alone or BEC activation as found by large-scale literature mining. These results validate adequate activation of hCMEC/D3 cells with inflammatory insults and suggest the validity of the model for further discovery of BEC-Th17 IIPs. 

The majority of the cellular, membrane, and secreted proteins identified (>75%) were not previously described to be associated with BEC, BBB, luminal or abluminal membranes, CNS inflammation or leukocyte trafficking; 50 of these molecules were surface adhesion molecules that have not been previously reported in BEC in response to inflammation. Thus, the use of advanced proteomic and glycoproteomic tools led to identification of a large number of novel molecules in the luminal glycocalyx and other cellular compartments of human BEC.

#### 6.2.2. Brain-Homing T Cells

The same advanced tools were also utilized to discover novel molecules in leukocytes. T cells with encephalo-tropism were generated by activation of lymphocytes, isolated from multiple sclerosis patients, with IL-23 as recently described [7]. These IL-17-producing T-helper (i.e., Th17) cells were utilized for further analysis, which included isolation of cellular and secreted proteins and performing label-free proteomics/glycoproteomics to identify molecular changes. More than 2850 cellular, 1875 membrane and 450 secreted proteins and glyoproteins were identified in the IL-23-activated Th17 cells. These included the key glycoproteins IL-17, IL-22, INF*γ*, and TNF*α* in the secreted fraction, validating the adequate activation of the cells. Well-known membrane glycoproteins involved in endothelium adhesion, such as LFA1, Mac1, and VLA4, were also detected in the activated Th17 cells. Furthermore, a number of proteins involved in *intra*cellular signaling and downstream pathways were also identified, including paxilin, talin, vinculin, Arp2/3, wasp, and cytoskeletal proteins. Since these proteins are involved in cell migration and projection—including formation of podosome-like structures for invasion—the Th17 cells appear to be primed for the anticipated attachment, adhesion, and diapedesis process. The majority of the molecules (>80%) identified in the activated Th17 cells (in both membrane and secreted fractions) were not described in the literature in association with T lymphocytes, inflammation, or leukocyte trafficking. These newly Th17-associated molecules include 10 additional integrins and more than 200 additional adhesion molecules, suggesting the potential importance of new molecular interactions and interacting pairs between Th17 and BEC.

**Figure 2:**
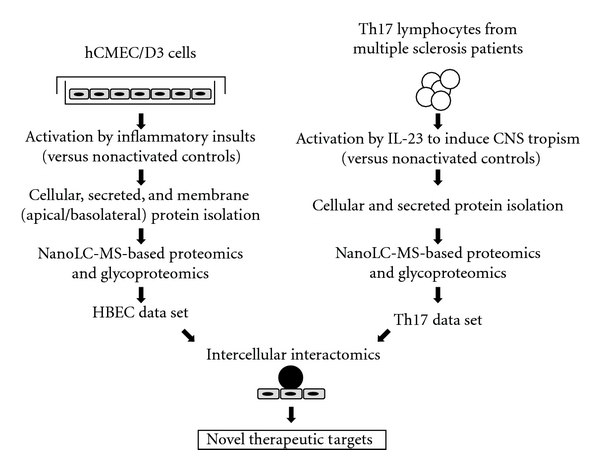
Schematic representation of the experimental approach used for described studies. Immortalized human brain endothelial cell line, hCMEC/D3 was exposed to various inflammatory cytokines or oxygen-glucose deprivation to identify differentially expressed proteins in response to these stimuli in luminal and abluminal membranes as well as in secreted proteins. Th17 lymphocytes derived from MS patients were exposed *in vitro* to IL-23 to induce their CNS tropism and to identify proteins regulated by this treatment. Respective databases of regulated proteins in inflammation-primed brain endothelial cells (BEC) and CNS-tropic Th17 lymphocytes were subjected to in sillico interactomics analyses to map putative protein-protein interactions that may contribute to Th17 cell adhesion and transmigration across the blood-brain barrier (BBB).

The summary of the experimental approach used to generate membrane and secreted proteome data sets from human BEC and brain-tropic Th17 lymphocytes is shown in Figure 2.

### 6.3. Phase 3: Constructing Intercellular Networks between hBEC and T Cells

**Figure 3:**
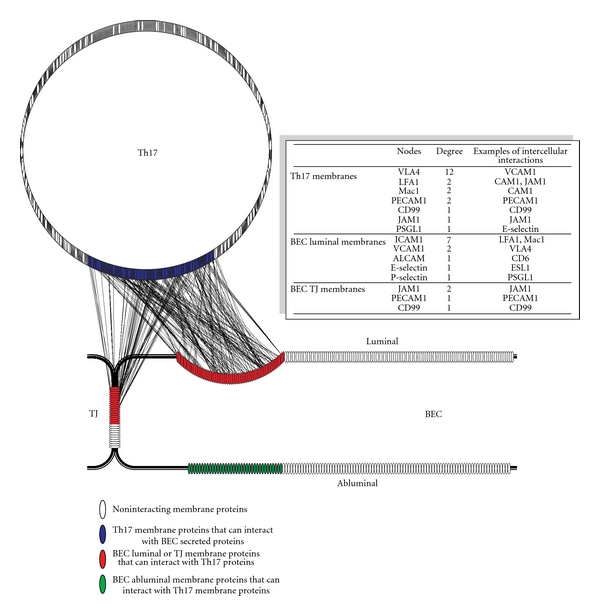
Shown is a visual depiction of the intercellular interaction network between proteins identified in Th17 lymphocytes and hCMEC/D3 brain endothelial cells using approach described in Figure 2. Each identified protein is represented by an oval; noninteracting proteins are shown in white, membrane proteins identified in Th17 lymphocytes that can interact with luminal membrane proteins of brain endothelial cells (BEC) are shown in blue, BEC luminal membrane- and tight junction proteins that can interact with Th17 membrane proteins are shown in red, and BEC abluminal proteins that can interact with Th17 membrane proteins are shown in green. Proteins were identified using membrane and subcellular proteomics and glycoproteomics and their interactions were catalogued using protein-protein databases as described in the text (each interaction is shown as a line connecting interacting proteins). *Insert*: the table shows a list of known proteins identified from the interactome network. Nodes represent the proteins or connection points in the membranes of Th17 and BEC in the network, and degree is the number of *intercellular* connections per node. Examples of these intercellular interactions are shown in the Table.

**Figure 4:**
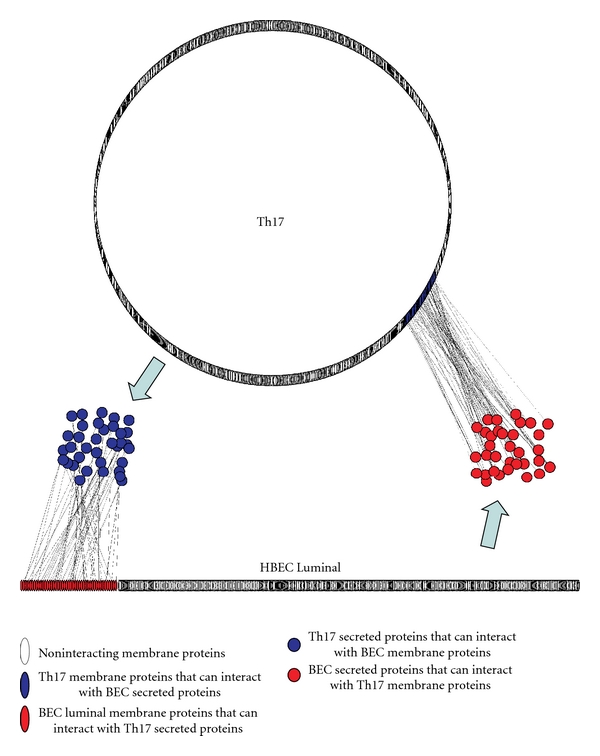
Extracellular signaling (communication) between Th17 and BEC. Shown is a visual depiction of the intercellular interaction network between proteins secreted by either Th17 lymphocytes or BEC and their interacting membrane counterparts expressed on the other cell type. All identified membrane proteins are represented by ovals, whereas secreted proteins are represented as circles: in red are luminal BEC membrane proteins that interact with secreted T17 proteins (in blue circles); in blue are membrane T17 proteins that interact with secreted BEC proteins (in red circles). Proteins were identified using membrane and subcellular proteomics and glycoproteomics and their interactions were catalogued using protein-protein databases as described in the text (each interaction is shown as a line connecting interacting proteins).

A computational approach was undertaken to construct intercellular interaction networks of stimuli-responsive molecules identified in human BEC and Th17 cells. This involved searching for each protein-protein interaction from the mPPI-db in the two proteomic datasets, such that one interactant is in the BEC and the other in the Th17 dataset. The resulting network consisted of more than 9000 interactions and was referred to as the master IIP network, since it represented all possible IIPs between the two datasets. However, most of these IIPs are unlikely to occur *in vivo*, since the intercellular pathological and physiological interactions and communications between T cells and BEC are limited to only accessible molecules, that is, surface and secreted molecules. To reduce the complexity of the master network and identify more relevant IIPs, we limited the interactions to cell-cell “*contacts*” and “*communications.*” The intercellular “contacts” consist of interactions between BEC and Th17 surface molecules, whereas intercellular “communications” consist of interactions between BEC secreted and Th17 surface molecules, and vice versa. For surface molecules, only proteins containing extracellular membrane domains were included. Furthermore, only molecules that were expressed under activated conditions were included. These criteria significantly reduced the master IIP network and produced a more relevant network of cell-cell contacts (Figure 3) and communications (Figure 4) between T cells and brain endothelium during leukocyte trafficking. 

### 6.4. Identification of Novel IIPs between Human BEC and T Cells

#### 6.4.1. Cell-Cell Contacts

A network of cell-cell contact points between human BEC and Th17 cells was generated to identify potential IIPs involved in the adhesion and diapedesis processes (Figure 3). The network shows interactions between molecules on membranes of Th17 with molecules on either luminal membranes or in the TJ of BEC. In addition, it shows the molecules on the abluminal membrane of BEC that can interact with Th17 surface molecules. It is apparent from the network (Figure 3) that many more IIPs are identified than there are currently known in the literature. Great number of molecules showed a high degree of interactions, that is, they interacted with more than one protein and with as many as 18 proteins. If accurate, these results suggest that interactions between T cells and the glycocalyx of endothelium are likely much more complex, involving a significantly larger number of molecules than previously believed.

More than 180 interacting pairs (IIPs) were detected between 116 Th17 membrane proteins and 62 human BEC glycocalyx proteins. About half of the molecules (55%) have been previously associated in the literature with endothelium or T-lymphocytes in general, but very few have been implicated specifically with either BBB, Th17 cells, or BEC-T cell adhesion/diapedesis. This suggests that many of the molecular changes in brain endothelium in response to inflammation are common with changes in other endothelia, and similar is true for Th17 and T cells. Some of identified IIPs between human BEC glycocalyx and Th17 membranes included VCAM1-VLA4, ICAM1-LFA1, ICAM1-Mac1, P-selectin-PSGL1, and E-selectin-ESL1, respectively (Figure 3, table insert). Most of the molecules involved in these IIPs had high number of intercellular interactions in the network. While connection points or “nodes” like these with high numbers of interactions in the network can potentially be used to locate potential targets to interrupt interaction between cells, more often they indicate very common, redundant, or nonselective interactions and consequently may lead to drug side effects [40]. The network however suggests that the remaining IIPs are novel and perhaps more specific to human BEC-Th17 interactions. 

Not unexpectedly, IIPs between human BEC TJs and Th17 membranes were also discovered (Figure 3), consistent with the postulated mechanisms of leukocyte diapedesis. During the paracellular diapedesis process, leukocytes may encounter homophilic interactions with TJ molecules, creating zipper-like contacts that replace the interendothelial junction [18]. Corroborating this and further validating the generated interactome network, we detected the known IIPs JAM1-LFA1, JAM1-JAM1, PECAM1-PECAM1, and CD99-CD99 pairs between the BEC TJs and the Th17 membranes, respectively. More than 50 additional IIPs between TJs and Th17 membranes were also identified, many of which have not been previously associated in this context. 

Finally, 113 IIPs between human BEC abluminal membranes and Th17 membranes were also identified (Figure 3). These are of significance since they might be involved in the diapedesis process through the transcellular pathway. In the final stages of this process, the transmigrating leukocytes need to transverse the abluminal membrane to enter the perivascular space and thus may recruit these IIPs.

Taken together, the cell-cell contact network provides new insights into the complexity of the adhesion and diapedesis processes and underscores that the intercellular interactions involved are likely not limited to just a few well-known IIPs, but rather extend to dozens of well-organized, cell-domain-localized molecular interactions. 

#### 6.4.2. Cell-Cell Communication

A network of cell-cell paracrine communications between human BEC and Th17 cells was also generated (Figure 4) to identify extracellular signals potentially involved in the cross-talks during leukocyte recruitment, adhesion, and diapedesis. The network shows the signals released from one cell which interact with their known receptors on the other cell's surface (Figure 4), depicting the potential cross-talk between leukocytes and the BBB during inflammation. Most of the signaling molecules identified were cytokines, chemokines, hormones, and/or growth factors. While the majority of the paracrine signaling molecules were not previously associated in the literature with leukocyte-BBB communication, several expected interacting pairs were also detected. These included MCP1, RANTES, and CCL19 cytokines in the BEC secretome and their respective receptors on the Th17 surface. Likewise, IL17, IL22, and others were detected in the Th17 secreted milieu and their receptors on the BEC glycocalyx. More than 25 additional extracellular signaling molecules from BEC were found to have receptors on Th17. Some of these receptors are known to be involved in T-cell recruitment/activation during the process of transmigration. Furthermore, more than 30 signals from Th17 were found to have receptors on human BEC, some of which are involved in receptor-mediated endocytosis/transcytosis. Overall, it was apparent that the paracrine communication between leukocytes and BBB under inflammatory conditions involves a large number of complex soluble ligand-receptor interactions which might lead to “priming” of the receptive cell for the anticipated diapedesis/transmigration process.

### 6.5. Phase 4: Validation of IIPs That Can Be Targeted Therapeutically

Validation of human BEC-T17 IIPs that may be suitable for the development of (blocking) therapeutic approaches could be done using various approaches. We propose that, after selection of potentially important interactions based on various bioinformatics algorithms that determine “strengths” of interactions in silico, potentially “drugable” interactions could be identified based on predetermined set of criteria including target molecule expression in brain vessels *in vivo*, accessibility from systemic compartment, brain/disease specificity, and so forth. In previous studies that compared the expression of proteins identified by 2D-gel and ICAT proteomics in BBB model(s) *in vitro* [38] and those identified using ICAT proteomics in laser-captured microvessels from animals *in vivo* [41], we identified and validated using immunochemistry and enzyme assays 19 commonly expressed proteins. In another proteomics study that identified more than 40 lipid-raft-specific proteins in human BEC *in vitro*, the expression of majority of these proteins has been confirmed in brain vessels in human brain tissue sections *in vivo* [26, 42, 43]. Most notably, activated leukocyte cell adhesion molecule (ALCAM) identified in these studies has been shown to promote leukocyte trafficking across the BBB through homotypic interaction with leukocytes; inhibition of this interaction reduced the severity and delayed the time of onset of experimental autoimmune encephalomyelitis in animal model. These studies provide validation that some of identified BEC-Th17 cell-cell interactions could be successfully targeted *in vivo* to inhibit leukocyte migration across the BBB. 

The systematic validation of the role of BEC-Th17 cell-cell interactions catalogued by methods described above in facilitating leukocyte transmigration could be achieved using a high-throughput antibody development approach that targets the relevant epitopes of interacting molecules. For example, this can be done using antibody display approaches (phage or ribosome display) or *in vivo* immunization against expressed epitopes of interacting molecules. Resulting antibody “libraries” could then be screened for antibodies with interaction blocking properties using various *in vitro* assays (Surface Plasmon Resonance—SPR, ELISA etc.) or biological readouts determine biological functions of the antibodies, for example, an efficacy in inhibiting leukocyte adhesion to brain endothelial cells or leukocyte transmigration across *in vitro* BBB models. Antibodies efficacious in modulating and inhibiting leukocyte adhesion and transmigration across the *in vitro* BBB model simultaneously validate the physiological or pathological role of identified IIPs. Identified “function-blocking” antibodies *in vitro*, could proceed into testing in various animal models, including EAE model [26]. The resulting lead molecule(s) could become amenable for further development as diagnostic, therapeutic, or theranostic for the treatment of neurological conditions accompanied, or caused by, leukocyte/lymphocyte infiltration. As a proof of concept for such an approach, we have generated a library of anti-ICAM-1 single-domain (VhH) antibodies from an immunized llama phage-displayed library, among which two antibodies displayed high affinity and ICAM-1 blocking activity in leukocyte adhesion assays (unpublished). One of these antibodies is currently being developed as molecular imaging agent for vascular inflammatory activation in stroke. Current advances in antibody engineering enable generation of bispecific antibodies that could simultaneously target more than one interacting molecule involved in the process of T-cell brain entry. 

The proof of concept for clinical translation of proposed approach has already been achieved with Natalizumab antibody, showing that the inhibition of leukocyte recruitment into the brain by antibody that blocks one of molecular interactions between leukocytes and BEC resulted in clinically successful control of inflammatory brain disease. The hypothesis and experimental approach described in this paper provide the opportunity to identify and clinically target other important leukocyte-BEC interactions that are more specific/selective for brain-tropic leukocyte subsets.

## 7. Conclusions

To develop new therapies for inhibiting CNS inflammation, there is a need to identify novel interacting molecules between CNS-homing T cells and activated BEC. Currently only a limited number of molecules on leukocytes and BEC are known or known to interact. We have hypothesized here, and have been successful in demonstrating, that interacting molecules of potential therapeutic significance can be discovered through the combination of advanced membrane/submembrane proteomic and interactomic methods. A number of novel protein-protein interactions identified between BEC luminal and Th17 membrane molecules by these methods are key targets for inhibiting various stages of T-cell entry into brain, including T cell/BEC attachment, firm adhesion, and diapedesis. In addition, formation of specialized domain, including recruitment of the “transmigratory cup” complex at luminal membranes in BEC and evidence of podosome-associated molecules on the activated T cells, not only suggests that the cells are preparing and ready for interactions, but also identifies these structures as important therapeutic targets. Furthermore, interactions identified between BEC tight junction and Th17 membrane molecules are key therapeutic targets for inhibiting the paracellular diapedesis route. The identified TJ molecules may also be involved in the transcellular process since Muller and coworkers recently showed that the membrane from a parajunctional reticulum of interconnected vesicles, containing JAM1, PECAM1, and CD99, is recruited to surround the transcellularly migrating leukocytes and aids in the process [44]. Interactions identified between BEC abluminal and Th17 membrane molecules are also interesting as therapeutic targets since they may be involved in the final stages of the diapedesis process (by either route). Finally, intercellular paracrine signaling between T cells and BEC is also considered an important target for inhibiting recruitment and activation of the leukocytes. Collectively, identification of novel human BEC-Th17 IIPs not only allows more in-depth understanding of the interactions and communications between CNS-homing T cells and activated BEC, but also allows discovery of novel targets for therapy at different stages of the CNS inflammation. 

The combination of advanced methodologies described here also has other applications. The use of emerging membrane/submembrane proteomic and glycoproteomic methods is applicable to other systems to allow identification of blood-accessible luminal and secreted targets. The intercellular interactomics has applications in understanding the complex interactions and communications among the various cell types in other systems. 

The significance of the molecules discovered by the advanced “omic” methodologies described here also goes beyond the scope of this hypothesis. Novel molecules identified on the luminal surfaces of the activated BEC are also attractive targets for molecular imaging-based diagnosis of inflammatory insults in the brain since they are blood-accessible. Molecules identified in IL-23 stimulated Th17 cells may clarify the mechanism of tropism of these cells toward the CNS. Furthermore, secreted signaling molecules from BEC and T cells also target other cell types, including cells in the neurovascular unit, as well as in the peripheral tissues. It would be interesting to further elaborate these intercellular networks to decipher the complex communications within the CNS, as well as between the CNS and the peripheral environment.
